# Illuminating compartmentalized AMPK signaling in single cells

**DOI:** 10.1042/BCJ20250267

**Published:** 2026-05-07

**Authors:** Arnav Jhawar, Kasey Parks, Danielle L. Schmitt

**Affiliations:** 1 Department of Chemistry and Biochemistry, University of California Los Angeles, Los Angeles, CA 90095, U.S.A.; 2 Institute for Quantitative and Computational Biosciences, University of California Los Angeles, Los Angeles, CA 90095, U.S.A.; 3 Molecular Biology Institute, University of California Los Angeles, Los Angeles, CA 90095, U.S.A.

**Keywords:** AMPK, biosensor, compartmentalized

## Abstract

AMP-activated protein kinase (AMPK) is a crucial regulator of cellular energy balance, affecting numerous downstream targets across various subcellular locations. Under cellular energy stress, AMPK becomes fully activated when it binds AMP and is phosphorylated by upstream kinases, including liver kinase B1 and calcium/calmodulin-dependent protein kinase kinase 2 (CaMKK2), and works to restore metabolic equilibrium. Additionally, CaMKK2 can activate AMPK independent of AMP in response to calcium signaling. In the present review, we summarize how genetically encoded kinase activity reporters for measuring AMPK activity have evolved for a comprehensive measurement of spatial and temporal AMPK activity in single cells. These reporters have provided important insights into AMPK activity dependent upon upstream kinases, location, and signaling cues. We also discuss the use of genetic actuators such as the AMPK inhibitory peptide that can be targeted to suppress AMPK activity at specific compartments. Together, these advances have established AMPK as a key regulator of metabolism with distinct spatial and temporal signaling patterns, suggesting compartmentalization of AMPK activity in the cell.

## Introduction

AMP-activated protein kinase (AMPK) is a crucial cellular energy sensor that coordinates metabolic responses by up-regulating catabolic processes and inhibiting anabolic processes under cellular stress to maintain energy homeostasis [1]. AMPK is an obligate heterotrimeric protein consisting of an α catalytic subunit, which contains the canonical kinase domain; a β regulatory subunit; and a γ nucleotide-binding subunit. In mammals, there are two isoforms of the α subunit, two isoforms of the β subunit, and three isoforms of the γ subunit, resulting in a minimum of 12 different AMPK isoforms, each with tissue-specific expression and potential implications in differential regulation [2]. Importantly, the γ subunit harbors four cystathionine β-synthase motifs, out of which three enable allosteric regulation of AMPK by the cellular energy charge, such that AMPK is inhibited by high levels of ATP, and activated by high levels of AMP [3]. AMP binding is thought to induce a conformational change to allow upstream activators of AMPK, canonically liver kinase B1 (LKB1) and calcium-calmodulin-dependent protein kinase kinase 2 (CaMKK2), to phosphorylate the activation loop harbored on the AMPKα subunit, resulting in maximal AMPK activity [4–9]. LKB1 was identified as the first upstream regulator of AMPK and is now known to regulate the larger AMPK family of kinases, which are all thought to phosphorylate similar peptide sequences but under different activating conditions [10]. Previous studies have also shown that CaMKK2 can activate AMPK in a γ-subunit-independent manner by only relying on intracellular calcium levels [11]. Several reports suggest that LKB1 is involved in activating AMPK in response to energy stress, while CaMKK2 is required for AMPK activity in response to hormone or growth factor signaling, although the regulation of AMPK by each kinase is likely cell-type dependent [12–15]. Once activated, AMPK broadly regulates processes to restore cellular energy homeostasis; thus, AMPK is often referred to as a ‘master regulator’ of metabolism.

There are over 100 downstream targets of AMPK identified, which are found in multiple subcellular compartments, including the cytoplasm, lysosome, mitochondria, endoplasmic reticulum, and nucleus [16,17]. For instance, acetyl-CoA carboxylase was the first identified downstream target of AMPK and is localized to the mitochondrial outer membrane and cytoplasm, while another AMPK target, HMG-CoA reductase, is localized to the endoplasmic reticulum [18]. Thus, an outstanding question in the field is: how is AMPK functioning throughout the cell in distinct compartments for efficient and effective regulation of cell function? Recent advances in our understanding of signal transduction have revealed that cell signaling is highly compartmentalized, with some signaling networks suggested to be organized in ‘signalosomes’ and many kinases identified as having distinct subcellular signaling environments [19,20]. For instance, the lysosome has been suggested to function as a signaling hub for AMPK under glucose starvation, where AMPK can coordinate with LKB1 using AXIN as a scaffold to phosphorylate several downstream effectors for precise and location-specific regulation of metabolism [21–24]. At the mitochondria, specific isoforms of AMPK, mainly α1, α2, β2, and γ1, have been suggested to localize to the outer mitochondrial membrane (OMM) and regulate mitophagy and mitochondrial quality control during exercise in skeletal muscle [25]. In the nucleus, AMPKα1 has been suggested to be selectively activated in response to genotoxic stress [26]. An emerging view is that AMPK activity in different compartments of the cell might have distinct impacts on cell function [27,28]. However, traditional approaches to study subcellular or compartmentalized AMPK signaling have relied on bulk assays, which can miss spatial and temporal dynamics of cell signaling, particularly at the single-cell level.

One such approach to studying AMPK activity that enables the capture of both spatial and temporal AMPK activity is the use of genetically encoded biosensors, referred to as kinase activity reporters (KARs). KARs are genetically encoded, typically fluorescent protein-based, reporters that, when expressed in living systems, report kinase events via changes in fluorescence [29]. In contrast to traditional bulk assays that are often static and endpoint, KARs enable visualization and quantification of continuous, real-time signaling events with high spatiotemporal resolution while preserving the native biochemical context [30]. KARs for AMPK consist of two domains: (1) the sensing domain, a substrate peptide that is phosphorylated by AMPK and a phosphoamino acid binding domain (PAABD) that binds the phosphorylated substrate peptide; and (2) a reporting domain, generally fluorescent proteins, although more recent advances use the self-labeling HaloTag [31,32]. When active, AMPK phosphorylates the substrate peptide, inducing PAABD binding to the phosphopeptide, inducing a conformational change that is propagated through to the reporter unit. This change in the fluorescent properties of the reporting unit gives a readout of kinase activity, enabling the visualization of real-time, quantifiable kinase signaling dynamics. In the present review, we provide a historical perspective on advances in developing KARs for AMPK, highlight key findings about spatial and temporal AMPK activity made using KARs, and discuss future applications for real-time measurement of AMPK activity across scales.

## AMPK activity reporters

### FRET-based AMPKARs

**Figure 1:**
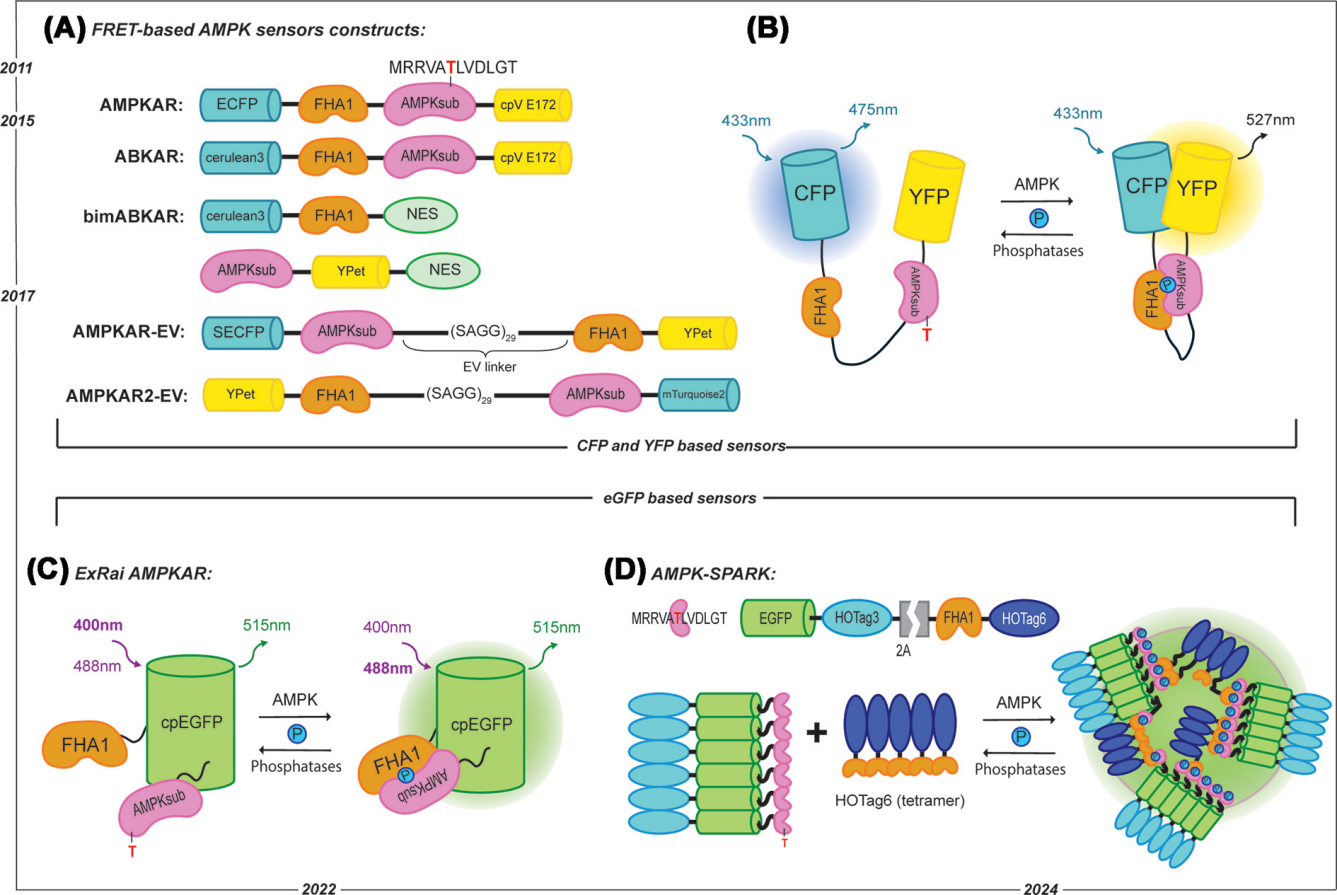
Design of genetically encoded kinase activity reporters for AMPK (AMPKARs) (**A**) Schematic diagram of design and domain structures of AMPKAR, ABKAR, bimABKAR, AMPKAR-EV, and AMPKAR2-EV. The AMPK substrate sequence is shown with the target threonine highlighted in red. (**B**) The design of FRET-based AMPKAR. The FRET pair cyan fluorescent protein (CFP) and yellow fluorescent protein (YFP) flank the phosphoamino acid-binding domain, FHA1, and an AMPK-specific substrate, with the threonine subject to phosphorylation highlighted. Upon AMPK phosphorylation of the biosensor, a phosphatase-reversible conformational change is induced that alters the FRET efficiency. (**C**) Design of excitation-ratiometric AMPKAR (ExRai AMPKAR), where circularly permutated EGFP (cpEGFP) is between the AMPK substrate and FHA1 domains. AMPK phosphorylation or phosphatase activity induces conformational changes in ExRai AMPKAR, resulting in switching of the excitation wavelength of cpEGFP from 400 nm when the biosensor is unphosphorylated to 480 nm when the biosensor is phosphorylated. (**D**) Design of phase-separation based AMPKAR (AMPK-SPARK), where the AMPK-specific substrate sequence, EGFP, and hexameric HOTag3 compose the first component. The tetrameric HOTag6 and the phosphoamino-binding domain, FHA1, sequence make up the second component. Phase separation enables the two components to cluster and create bright fluorescent droplets once AMPK is active. In contrast, phosphatases function to break up the clusters that have formed.

Tsou et al*.* developed the first AMPK activity reporter (AMPKAR), which enables measurement of AMPK dynamics in real time. Adapted from a previously developed KAR for protein kinase A (PKA), AMPKAR contains an optimized AMPK substrate motif and a PAABD specific for phosphothreonine, the forkhead-associated domain 1 (FHA1) sandwiched between the Förster resonance energy transfer (FRET) pair of eCFP (donor) and a circularly permutated variant of Venus cpV E172 (acceptor; Figure 1A,B) [33]. To identify the optimal AMPK substrate, the authors screened a positional peptide library, revealing that AMPK showed a strong preference for Leu at +4, Met or Leu at −5, and Arg at −3 or −4. Leu at −5 was a potential phosphorylation site for protein kinase D family members, a group with selectivity like that of AMPK. After testing multiple substrate motifs, they observed an optimal dynamic range with MRRVATLVDL, where AMPK phosphorylates Thr, and moved forward with this sequence for AMPKAR. When a FRET pair with a sufficient spectral overlap is within proximity (<10 nm) with favorable dipole-dipole alignment, the donor fluorescent protein nonradiatively transfers energy to the acceptor fluorescent protein, resulting in FRET [30,34]. This leads to quenched donor emission and a sensitized acceptor emission, which can be measured as FRET. Upon phosphorylation of the AMPK substrate motif, FHA1 binds the phosphopeptide, bringing the FRET pair closer together and increasing the yellow FP (YFP) to cyan FP (CFP) emission ratio, or FRET ratio [33].

To study localized AMPK activity, AMPKAR was fused to either a nuclear localization signal (NLS) or a nuclear export signal (NES) (Table 1). Subcellular localization of AMPKAR allowed for measurement of spatial AMPK activity with temporal resolution. Energy stress induced by inhibition of glucose metabolism using 2-deoxyglucose (2-DG) mainly activated AMPK in the cytosol with minimal measured nuclear AMPK activity, while Ca^2+^-induced AMPK activity using ionomycin was measured in both the cytosol and the nucleus. This seminal work, developing the first AMPK biosensor, revealed real-time spatial AMPK activity under energy stress and motivated further study of subcellular AMPK activity.

**Table 1:** Summary of all AMPK reporters and subcellular locations targeted

Reporter	Localization	Citation
AMPKAR	Untargeted and nucleus	[33]
ABKAR	Untargeted, cytoplasm, nucleus, plasma membrane, Golgi apparatus, endoplasmic reticulum, mitochondria, and lysosome	[37,38]
bimABKAR	Cytoplasm and plasma membrane	[41]
AMPKAR-EV	Untargeted	[42]
AMPKAR2-EV	Untargeted	[43]
ExRai AMPKAR	Untargeted, nucleus, mitochondria, and lysosome	Schmitt et al.
AMPK-SPARK	Untargeted	[56]
AMPfret	Untargeted	[58]

This first-generation AMPKAR suffered from some of the technical limitations of FRET-based sensors. These biosensors demonstrate relatively modest dynamic range and diminished sensitivity when used to track kinase activity [35,36]. One approach to overcome this challenge is to optimize the fluorescent proteins used in the biosensor, selecting a FRET pair with enhanced FRET efficiency. Sample et al. replaced ECFP from AMPKAR with a brighter cyan variant, Cerlulean3 (Figure 1A) [37]. This improved AMPKAR exhibited a two-fold increase in response upon 2-DG treatment. When using this improved KAR in neurons, it was found that the related AMPK family members, brain-specific kinases 1 and 2 (BRSK1 and BRSK2), can phosphorylate the biosensor. Thus, Sample et al*.* termed their reporter AMPK and BRSK dual-activity reporter (ABKAR). Using ABKAR, the difference in spatial dynamics between AMPK and BRSK was illuminated. Energy stress induced by 2-DG or Ca^2+^ stimulation activated AMPK across the whole neuron, with the strongest response in the distal axon. Using neurons with BRSK1/2 knocked down revealed that there is high basal BRSK1/2 activity in the axon of polarized neurons, and BRSK1/2 is not responsive to Ca^2+^ stimulation or energy stress.

In subsequent work, Miyamoto et al. used ABKAR to profile spatial AMPK activity in mouse embryonic fibroblasts (MEFs), demonstrating compartmentalized AMPK activity at subcellular locations like the mitochondria and lysosome [38]. This was accomplished by fusion of ABKAR to proteins or peptide sequences that localize to the cytosolic surface of various cellular compartments. They utilized well-established localization motifs, including an N-terminal myristylation motif from Lyn kinase for the plasma membrane, NOS3 for the Golgi, CYP2C1 for the ER, AKAP1 for the mitochondria, LAMP1 for the lysosome, and SV40 large T antigen for nuclear localization, with localization validated by compartment-specific markers before functional imaging (Table 1). As the fluorophores, substrate motif, and phospho-binding domain were identical across the panel, any differences in kinetics or amplitude could be attributed more precisely to location rather than to sensor architecture.

Across all endomembrane compartments, AMPK exhibited higher activity in wild-type MEFs than in AMPKα1/2 knockout (α1/2 KO) MEFs in response to 2-DG or glucose starvation, suggesting that AMPK is activated under energy stress across all subcellular locations. Miyamoto et al. found that metabolic perturbations, like 2-DG or glucose starvation, generate distinct compartment-specific AMPK patterns. Glucose starvation resulted in early AMPK activation at the plasma membrane, Golgi, and the endoplasmic reticulum (ER), with delayed kinetics at the mitochondria and the lysosome, whereas glycolysis inhibition via 2-DG exhibited rapid activation at all compartments except for the mitochondria, where sustained activity was noticed after 2 h. Surprisingly, metabolic perturbation showed minimal AMPK activity at the nucleus even after 4 h. The authors proposed that distinct metabolic profiles could encode input-specific propagation patterns, and they also assessed the location-specific differences in AMPKα isoforms, finding that α1 reconstitution, rather than α2, preferentially restored AMPK activity at the plasma membrane and exhibited a higher FRET signal in the Golgi apparatus and lysosome [38].

Another challenge is ensuring that the sensing domain traverses conformations within the appropriate distance and orientation ranges to maximize the dynamic range of the FRET ratio [39,40]. One approach to increasing the dynamic range of FRET-based KARs is to decrease the level of basal or initial FRET ratio in unstimulated conditions by increasing the distance between the FRET pair. To produce a reporter with high dynamic range, Depry et al. generated a bimolecular kinase activity reporter where the traditional unimolecular KAR design was split in half, such that the substrate peptide and PAABD are each fused to one half of a FRET pair, and kinase phosphorylation of the substrate peptide induces PAABD binding and an increase in FRET ratio. ABKAR was engineered for this bimolecular design, and the previous FRET pair was replaced with Cerulean and YPET, generating bimolecular ABKAR (bimABKAR; Figure 1A and Table 1) [41]. When localized to the cytoplasm, bimABKAR had a similar dynamic range as ABKAR in response to 2-DG but slower kinetics, presumably due to diffusion in the cytoplasm. However, when localized to the plasma membrane by fusion of bimABKAR components to the membrane targeting motif from KRAS, bimABKAR outperformed ABKAR in cells treated with 2-DG, enabling more-sensitive detection of AMPK activity at the plasma membrane. Using plasma membrane-targeted bimABKAR, Depry et al*.* uncovered the bidirectional regulation of AMPK activity at the plasma membrane by PKA.

Working to address limitations of FRET-based biosensors, Komatsu et al. took a two-step approach to develop a unimolecular biosensor with a higher dynamic range for imaging kinase activity. They first optimized the donor and acceptor FPs by replacing the existing CFP-YFP pair with a variety of teal fluorescent proteins (TFPs), CFP- and YFP-derived donor and acceptor pairs. Addressing the distance between the FRET pair, Komatsu et al*.* engineered a glycine-rich Eevee (EV) linker consisting of repeated SAGG motifs into KARs to lower the basal or initial FRET ratio, resulting in a larger dynamic range [40]. Leveraging these principles, Konagaya et al. developed AMPKAR-EV (Figure 1A), following the original naming convention [42]. AMPKAR-EV consisted of the extended EV linker and a FRET pair consisting of super-enhanced CFP and YPET and had a remarkably lower basal FRET ratio due to decreased EV linker-led association between the FPs in the absence of substrate phosphorylation. Taking advantage of the enhanced dynamic range of AMPKAR-EV, Konagaya et al. confirmed that LKB1 can phosphorylate and activate AMPK even in a nutrient-rich culture medium. AMPKAR-EV was the first AMPK sensor used *in vivo*, with transgenic mice stably expressing the sensor used to characterize the pharmacodynamics of AMPK activators and inhibitors across various tissue types. Metformin activated AMPK faster in hepatocytes than muscle fibers, attributed to the higher presence of the transporter used for metformin uptake, organic cation transporter 1, in the liver. The present work established a sensitive, broadly deployable tool to measure subtle changes in AMPK dynamics in live cells and intact tissues, setting the stage for more complex, spatially targeted AMPK reporters.

Building off the evolution of the EV linker, Hung et al*.* developed AMPKAR2-EV, which still retains the EV linker and instead uses mTurquoise2 and YPET as the FRET pair (Figure 1A) [43]. With AMPKAR2-EV, long-term (20-h) imaging of AMPK activity was accomplished. Hung et al*.* investigated the cross talk between AMPK and Akt using KARs for both kinases, finding that AMPK and Akt exhibit oscillatory behaviors over time in response to inhibition of glycolysis. Notably, these AMPK-Akt oscillations are asynchronous. When AMPK activity was high, Akt activity was low, and conversely, when Akt activity was high, AMPK activity was low. Thus, through measuring long-term dynamics of metabolic signaling networks in single cells using KARs, the tight temporal regulation of cell function could be uncovered.

### Single fluorophore-based AMPKARs

While FRET-based AMPKARs significantly advanced our understanding of real-time and compartmentalized AMPK dynamics, FRET-based biosensors can suffer from poor dynamic range and limited multiplexing capabilities, as C-Y FRET occupies a large portion of the visible light spectrum. Single-fluorophore sensor designs, however, often occupy less of the visible light spectrum, making multiplexing easier, resulting in the ability to measure multiple cellular activities at once [44]. In general, this sensor design tends to deliver higher dynamic range and sensitivity, detecting subtle activity changes and tracking multiple signaling events in the same cell, something many FRET workflows struggle with in practice [45].

To better elucidate AMPK activity within the cell, we developed a single fluorophore excitation-ratiometric AMPKAR (ExRai AMPKAR). ExRai AMPKAR consists of the optimized AMPK substrate motif fused to a cpEGFP and FHA1 (Figure 1C). When ExRai AMPKAR is dephosphorylated, the cpEGFP prefers a 400 nm excitation, but when AMPK is activated and phosphorylates the substrate peptide, FHA1 binding induces a conformational change in the biosensor that shifts the preferred excitation wavelength of cpEGFP to 480 nm, while the emission remains constant at 520 nm. ExRai AMPKAR reports AMPK activity via a ratiometric readout of the excitation-emission (480 nm excitation-emission/400 nm excitation-emission). ExRai AMPKAR had a three-fold higher response in comparison with previous FRET-based reporters, making it the most sensitive AMPKAR developed to date. ExRai AMPKAR was found to have a better assay Z-factor score than ABKAR, indicating a robust and reproducible biosensor [45].

**Figure 2:**
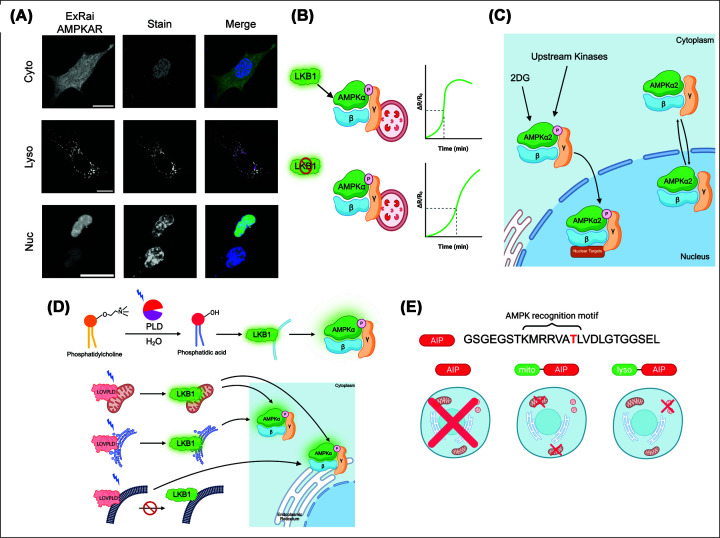
Visualizing spatiotemporal AMPK regulation across compartments using ExRai AMPKAR and AMPK inhibitory peptide (AIP) (**A**) (From top to bottom) Representative image of ExRai AMPKAR in MEFs stained with a DAPI nuclear stain; outer lysosomal membrane-localized ExRai AMPKAR (lyso-ExRai AMPKAR) in MEFs stained with the lysosomal marker Lyso Tracker Red; and nuclear-localized ExRai AMPKAR (NLS-ExRai AMPKAR) in MEFs stained with a DAPI nuclear stain. Scale bars represent 20 um. (**B**) Upstream kinase LKB1 phosphorylates AMPK, leading to faster kinetics at the lysosome. Under LKB1 deletion, lysosomal activity maintains the same response amplitude but looses its kinetic advantage. (**C**) Mechanism of 2-DG-induced nuclear AMPK activity, where nuclear AMPK activity in response to 2-DG is initiated in the cytoplasm dependent on upstream kinases, after which AMPK then translocates into the nucleus to phosphorylate nuclear targets. (**D**) Phosphatidylcholine can be converted to phosphatidic acid via blue light illumination on LOVPLD. PA helps recruit LKB1 to membranes, in turn activating AMPK. Organelle-confined PA generation recruited GFP-LKB1 to the mitochondria and the endoplasmic reticulum but not the plasma membrane. PA production at these sites led to activation of AMPK at multiple sites irrespective of LKB1 recruitment, indicating organelle cross-talk and metabolite diffusion. (**E**) AIP sequence with the target threonine highlighted in red. AIP when expressed without any localization sequence inhibits total AMPK activity. AIP can also be targeted to other compartments such as the mitochondria and the lysosome.

Using previously established targeting approaches, we localized ExRai AMPKAR to the OMM, lysosomal surface, and nucleus by appending known localization motifs for each location to the biosensor (Figure 2A and Table 1). We measured distinct spatiotemporal dynamics across compartments in response to energy deprivation, allosteric activation, and oxidative stress, and, across all perturbations, observed distinct spatiotemporal dynamics with AMPK rapidly activated at the lysosome and mitochondria and slower in the cytoplasm and nucleus [45,46]. These results confirmed previous studies that established the lysosome and mitochondria as key metabolic sensing and stress signal integration sites [47,48].

We then used the sensor to define upstream kinase wiring across all compartments. When AMPK was activated by either 2-DG or allosteric activation using MK-8722, a molecule that binds to the allosteric drug and metabolite site on AMPK, LKB1 deletion suppressed cytoplasmic AMPK activity, while there was no significant difference in the amplitude response at the lysosome or the mitochondria. Instead, loss of LKB1 selectively impacted the lysosome's kinetic advantage by decreasing the rate of AMPK activation at the lysosome, suggesting LKB1 influences AMPK kinetics at the lysosome (Figure 2B) [22]. Notably, the enhanced sensitivity of ExRai AMPKAR enabled robust measurement of AMPK activity in the nucleus, which previous FRET-based AMPKARs were unable to do. Using ExRai AMPKAR, we found that in AMPKα1/2 KO MEFs, nuclear-localized AMPKα2 was unable to rescue nuclear AMPK activity in these cells, whereas when AMPKα2 can translocate between the cytoplasm and nucleus, nuclear activity was restored. Thus, using ExRai AMPKAR, we established a mechanism for nuclear AMPK activity dependent on the translocation of activated AMPK from the cytoplasm to the nucleus (Figure 2C) [45].

Li et al*.* employed ExRai AMPKAR to determine whether acute, organelle-confined phosphatidic acid (PA) production drives AMPK activation via LKB1 recruitment. PA is a multifunctional lipid that participates in major signaling pathways, including Hippo and LKB1-AMPK [49–51]. It does this by serving as a membrane anchor to recruit protein complexes at specific organelle membranes, thus enabling localized signaling and cell metabolism [52,53]. The authors leveraged an ontogenetically gated phospholipase D (PLD; LOVPLD), which, when exposed to blue light, can generate PA. This construct could be organelle-targeted for local PA production (Figure 2D) [54].

Li et al*.* first found that PA production at several organelles could recruit GFP-LKB1 from the nucleus to those membranes, except the plasma membrane. Since recruitment of LKB1 alone is not an effective readout of AMPK activation, they deployed ExRai AMPKAR in the cytosol and the cytosolic leaflet of the ER to measure local AMPK activity. Upon illumination, mitochondrial LOVPLD increased AMPK activity in both the cytosol and the ER. ER-localized LOVPLD increased cytosolic AMPK activity, and plasma membrane LOVPLD increased ER AMPK activity, despite the lack of detectable LKB1 recruitment to the plasma membrane (Figure 2D). This pattern suggested a model in which organelle-generated PA can activate AMPK through inter-organelle cross-talk and diffusion, rather than a simple model in which local PA production recruits LKB1 and activates AMPK at the same site [54]. Thus, single-fluorophore reporters like ExRai AMPKAR enabled compartment-specific AMPK measurements with improved dynamic range, sensitivity, and spatiotemporal resolution.

### Phase separation-based AMPKAR

Another approach for multiplexed imaging of AMPK activity leverages the separation-of-phases-based activity reporter (SPARK) design construct, AMPK-SPARK [55]. SPARK-based KARs rely on kinase-induced multivalent interactions that drive protein phase separation and formation of droplets. The design of AMPK-SPARK consisted of two components. First, the optimized AMPK substrate motif is fused to an EGFP and a homo-oligomeric tag3 (HOTag3), a multivalent homo-oligomeric coiled coil peptide. Second, FHA1 is fused to another coiled-coil domain, HOTag6. These components were linked on the same plasmid by a self-cleavable T2A sequence, allowing co-expression of both components at approximately a 1:1 ratio. Upon AMPK activation, FHA1 binds the now phosphorylated AMPK substrate motif, resulting in multivalent interactions driving phase separation, as observed by puncta in the cell (Figure 1D) [56].

For multiplexed sensing of both AMPK activity and calcium dynamics with the same biosensor, the calcium biosensor GCaMP6f was put in place of the EGFP [57]. An increase in cytosolic fluorescence reflected a rise in the intracellular calcium levels, while cluster formation indicated AMPK activity. Using this new design, Erdoğan et al*.* were able to understand the duration, persistence, and scale of calcium and AMPK dynamics together in a single cell using one biosensor. Different AMPK activation patterns were observed across cells when comparing calcium elevation and energy stress conditions, suggesting a greater relevance of context-specific AMPK regulation. A whole-cell population showed similar calcium elevation dynamics but exhibited heterogeneity in AMPK activation. For instance, glucose starvation resulted in an enhancement in AMPK-SPARK puncta formation, but no change in overall fluorescence. When glucose starvation was followed by 2,5-di(tert-butyl)-1,4-hydroquinone to increase intracellular calcium levels, both an increase in AMPK-SPARK puncta and overall cell fluorescence was measured. AMPK-SPARK has a high dynamic range, making it a suitable candidate for high-throughput drug screening and tissue applications. However, AMPK-SPARK has limited spatial resolution, making the biosensor best for whole-cell readout of AMPK activity [56].

### AMPK activation reporter

A unique way to understand kinase activation is to use the kinase itself as a biosensor. AMPfret, an AMPK activation reporter, was constructed by linking a CFP at the C-terminus of the AMPKα2 subunit, while a YFP was added to the C-terminus of the AMPKγ1 or AMPKβ2 subunit. Thus, through the inclusion of a CFP/YFP FRET pair, conformational changes in AMPK result in a change in measured FRET ratio. Upon AMP/ADP binding to the γ-CBS site, there is a shift in the FRET ratio, with ATP acting as a competitive ligand. This sensor, based on conformational changes in AMPK itself, can be used to measure relative ATP, ADP, and AMP concentrations within the cell. Pelosse et al. used AMPfret *in vitro* to dissect how individual CBS sites in the γ subunit contribute to conformational changes in AMPK in response to fluctuations in the cellular energy charge [58]. In cells, AMPfret was used to measure AMPK activation in response to allosteric activation using AICAR or energy stress induced by 2-DG, finding that the response to AICAR was more rapid than 2-DG as AICAR functions as an allosteric activator rather than manipulating cellular metabolism. However, AMPfret has some limitations in the modes of AMPK regulation AMPfret can detect. For instance, Palmitoyl-CoA can allosterically activate AMPK-β1-containing complexes, which cannot be measured using the currently existing AMPfret design, which consists of α2β2γ1. Additionally, CaMKK2 can activate AMPK independent of AMP, which limits the application of AMPfret to study the complex interplay of AMPK and calcium-mediated signaling through CaMKK2 [11,18]. Thus, AMPfret enables a direct readout of AMPK activation via conformational change of the kinase itself.

### Manipulating AMPK activity

While AMPKARs and related tools like AMPfret enable direct visualization of AMPK activity with high spatiotemporal resolution, complementary approaches that perturb AMPK signaling in a compartment-restricted manner enable mechanistic determination of the role of spatial AMPK activity in cell function. Genetically encoded AIPs functions as competitive pseudosubstrates to suppress the catalytic activity of AMPK (Figure 2E). Unlike small-molecule inhibitors, AIPs can be targeted to defined subcellular locations, enabling selective inhibition of local AMPK pools while largely preserving global kinase signaling.

The first implementation of the AIP was described by Miyamoto et al., who developed the AIP, using a short peptide derived from an AMPK substrate recognition motif that effectively suppressed AMPK-dependent phosphorylation events in cells. When expressed in mammalian cells, AIP inhibited phosphorylation of canonical AMPK substrates without altering upstream AMPK activation, consistent with a mechanism in which the peptide functions as a competitive inhibitor of substrate binding rather than blocking kinase activation. Importantly, this work established that AIP expression does not globally disrupt cellular energy status, allowing AMPK-dependent outputs to be selectively interrogated downstream of kinase activation [38].

AIP-based inhibition has proven particularly valuable when used in conjunction with genetically encoded AMPKARs. Miyamoto et al. first demonstrated this strategy by combining ABKAR with a mitochondria-targeted AIP construct. Using mitochondrial-localized ABKAR, they observed that constitutive localization of AIP to the mitochondrial surface selectively suppressed AMPK activity in this compartment without abolishing kinase activation at other locations. These experiments provided some of the earliest functional evidence that AMPK activity is spatially heterogeneous and can be selectively manipulated at defined organelles. This combined approach of using biosensors to report activity and AIPs to impose localized inhibition enables the detection of compartmentalized AMPK activity.

Building on this framework, AIP was later adapted as a genetically encoded tool to probe spatially restricted AMPK signaling. Drake et al*.* leveraged targeted AIP targeted to the mitochondria to find that inhibition of mitochondrial AMPK leads to oxidative stress. In mice expressing both mitochondrial-targeted AIP and the mitophagy biosensor MitoTimer in the flexor digitorum brevis muscle, local inhibition of AMPK resulted in suppressed exercise-induced mitophagy, suggesting AMPK is involved in regulating mitophagy induced by mitochondrial energetic stress. These findings provided direct functional evidence that compartmentalized AMPK activity contributes to distinct cellular responses in cells and *in vivo* [25].

Despite these advantages, several limitations of AIP-based approaches warrant consideration. First, because AIP acts as a competitive inhibitor, its efficacy depends on relative expression levels and local substrate concentrations, which may vary across compartments and experimental systems. Second, AIP inhibits AMPK catalytic activity broadly within its targeted locale but does not discriminate between AMPK complexes containing different regulatory subunits, potentially masking isoform-specific effects. Finally, unlike biosensors, AIP does not provide temporal information about AMPK activation dynamics, instead offering a static suppression of kinase output. Still, AIP remains a uniquely versatile genetic tool for dissecting compartmentalized AMPK signaling. When combined with biosensors, the AIP enables complementary gain- and loss-of-function interrogation of AMPK activity with spatial and temporal resolution. Together, these approaches have begun to reveal how AMPK integrates energetic stress signals in a spatially organized manner to coordinate diverse cellular processes.

## Conclusions and future opportunities

Over the last decade, our understanding of the role of AMPK in metabolism has shifted from viewing AMPK as a bulk cytosolic stress kinase to recognizing AMPK as a nexus in a spatially organized signaling network. Distinct AMPK pools are regulated by isoform diversity, local metabolic signals, and tissue-specific responses, which together explain compartment-specific downstream effects. Genetically encoded AMPK biosensors and organelle-targeted inhibitory peptides have been central to this conceptual shift, as they enable real-time visualization and perturbation of AMPK activity with spatial and temporal precision.

These studies have established practical standards for both targeted and non-targeted reporter design. During sensor development, key validation features, such as specificity controls, non-phosphorylatable mutants, and AMPK loss-of-function backgrounds, should be incorporated to ensure that any change in KAR response reflects AMPK activity rather than optical or localization artifacts [38]. Organelle-targeted biosensors typically have a straightforward design in which a localization sequence is fused to the sensor, facilitating the generation of multiple compartment-specific variants. However, this modular design makes proper validation and careful interpretation of compartment signals essential [59]. Maintaining moderate expression levels is also necessary to reduce mislocalization and buffering effects, even when basal signal intensity does not strongly correlate with expression levels [60,61].

Multiplexing needs drove the field to move from classic two-color FRET to single-channel and single-fluorophore designs. Because AMPK sits at the intersection of multiple upstream inputs, multiplexed approaches are particularly valuable to better deconvolve AMPK signaling networks. For example, simultaneous imaging of AMPK with calcium and energy-state reporters can distinguish CaMKK2-linked activation from activation driven by cellular ATP depletion. However, most live-cell metabolite and kinase biosensors operate in the same CFP/YFP FRET or GFP spectral range, limiting the ability to measure AMPK activity alongside reporters of glucose, ATP:ADP ratio, glycolytic intermediates, or NADH. A practical solution to this problem is the use of red-shifted fluorescent proteins or HaloTag-based KARs, which have already been developed for calcium and other kinases [32,62,63]. Additionally, as blue- and red-shifted biosensors for other cellular activities become more widely available, multiplexing of AMPK activity with measurements of other cellular activities will be able to uncover compartmentalized regulation of metabolism and cell function [64,65].

Distinct AMPK pools differ in activation timing, amplitude, and upstream kinase based on local cues. Genetically encoded tools have enabled us to test these varied patterns with high compartment and spatiotemporal resolution. Early on, AMPKAR imaging showed that energy stress and calcium inputs produce distinct spatial signatures at the cytosol and the nucleus and that single-cell dynamics are rather heterogeneous than uniform across populations [33]. Subsequent development of other reporters has helped strengthen the spatial argument in different ways. ABKAR imaging in neurons highlighted polarized activity patterns, and appending known localization sequences helped generate a panel of variants, showing that, superficially, the same glycolytic perturbations still yielded varied dynamic ranges [37,38]. Additionally, activation reporters like AMPfret allow for a precise understanding of allosteric regulation of AMPK activity, which can enrich our understanding of how the spatial regulation of AMPK activity is influenced by the local environment.

Current and future work improving AMPK reporters can push measurements of compartmentalized AMPK activity beyond cultured cells and towards intact tissues and whole animals. These approaches can help us answer specific questions about isoform specificity, spatial integration at organelle contact sites, and varied spatial AMPK signaling dynamics across tissue types. Future development of AMPKARs with higher sensitivity, a wider dynamic range, an improved signal-to-noise ratio, and a low background signal can broaden the range of targets AMPK reporters can interrogate and provide more insights into the spatiotemporal regulation modalities of AMPK. Thus, through the development of biosensors of AMPK, we have been able to illuminate the compartmentalized activity of this ‘master regulator of metabolism.’

## Open Access

Open access for this article was enabled through an open access agreement between the Biochemical Society/Portland Press and the University of California.

## Abbreviations

2-DG: 2-deoxyglucose
BRSK: Brain-selective kinase
ABKAR: AMPK/BRSK kinase activity reporter
AMPKAR: AMPK activity reporter
AIP: AMPK inhibitory peptide
AMPK: AMP-activated protein kinase
bimABAKAR: bimolecular AMPK/BRSK kinase activity reporter
CaMKK2: calcium/calmodulin-dependent protein kinase kinase 2
CFP: cyan fluorescent protein
cpEGFP: circularly permutated enhanced green fluorescent protein
ER: endoplasmic reticulum
EV: Eevee
FHA1: forkhead-associated domain 1
FRET: Förster resonance energy transfer
HOTag3: homo-oligomeric tag3
HOTag6: homo-oligomeric tag6
KARs: kinase activity reporters
LKB1: liver kinase B1
LOVPLD: Light-oxygen-voltage sensing domain phospholipase D
MEFs: mouse embryonic fibroblasts
NES: Nuclear export signal
NLS: nuclear localization signal
OMM: outer mitochondrial membrane
PA: Phosphatidic acid
PAABD: phosphoamino acid binding domain
PKA: protein kinase A
PLD: phospholipase D
SPARK: separation-of-phases-based activity reporter
TFP: teal fluorescent protein
YFP: yellow fluorescent protein
